# A Fast Intrusion Detection Method for High-Speed Railway Clearance Based on Low-Cost Embedded GPUs

**DOI:** 10.3390/s21217279

**Published:** 2021-11-01

**Authors:** Yao Wang, Peizhi Yu

**Affiliations:** 1 School of Mechanical, Electronic and Control Engineering, Beijing Jiaotong University, Beijing 100044, China; wangyao@bjtu.edu.cn; 2 Electrical and Computer Engineering, Carnegie Mellon University, Pittsburgh, PA 15213, USA

**Keywords:** intrusion detection, SSD, improved MobileNet, feature fusion module, embedded GPU

## Abstract

The efficiency and the effectiveness of railway intrusion detection are crucial to the safety of railway transportation. Most current methods of railway intrusion detection or obstacle detection are inappropriate for large-scale applications due to their high cost or limited coverage. In this study, we present a fast and low-cost solution to intrusion detection of high-speed railways. As the solution to heavy computational burdens in the current convolutional-neural-network-based detection methods, the proposed method is mainly a novel neural network based on the SSD framework, which includes a feature extractor using an improved MobileNet and a lightweight and efficient feature fusion module. In addition, aiming to improve the detection accuracy of small objects, the feature map weights are introduced through convolution operation to fuse features at different scales. TensorRT is employed to optimize and deploy the proposed network in the low-cost embedded GPU platform, NVIDIA Jetson TX2, to enhance the efficiency. The experimental results show that the proposed methods achieved 89% mAP on the railway intrusion detection dataset, and the average processing time for a single frame was 38.6 ms on the Jetson TX2 module, which satisfies the need of real-time processing.

## 1. Introduction

The rapid development of railway transportation has led to more and more attention to railway safety. It is greatly significant to ensure the safety of high-speed railway operations. Any obstacles or pedestrians intruding the railway track area are the major hazards to the safety and the security of the railway operations. Although, numerous intrusion detection systems with various types of sensors, e.g., laser scanners, infrared sensors, radars, optical fibers, etc., have been proposed in previous works, each specific type of sensor has its own drawbacks [1]. Recently, the machine-vision-based intrusion detection methods have been increasingly popular, benefiting from the rapid development of deep learning [2,3,4,5,6,7,8,9,10,11]. However, most early vision-based methods detect intruding objects with background subtraction methods, which have very poor detection effects due to lighting conditions or bad weather. In recent years, the convolutional neural networks (CNNs) have achieved promising results in visual tasks such as image classification, image segmentation, and object detection for its benefits of strong comprehensiveness, activeness, and its capability of detecting and identifying multiple types of objects simultaneously.

At present, the CNN-based object detection algorithm has been introduced to detect intruding objects, which can be categorized into two major types. The first one follows the traditional object-detection principles (such as R-CNN [12], fast R-CNN [13], and faster R-CNN [14]), which generate region proposals then classify each proposal into different object categories. Yu et al. [15] proposed a convolutional neural network feature extractor based on the faster R-CNN framework by using residual units to overcome the difficulty of detection of obstacles in the outdoor railway scene. The second type regards object detection as a regression or classification problem (such as YOLO [16] and SSD [17]) and adopts a unified framework to achieve final results (categories and locations) directly. Xu et al. [18] presented a method based on single shot multibox detection (SSD) to identify obstacles on rails. This algorithm generated multi-scale feature maps based on the residual neural network and added a series of convolution layers for feature fusion. Ye et al. [19] proposed an object detection system for railways that focused on detecting objects ahead of the train in the shunting mode. Detectors based on CNNs have achieved remarkable performance in the detection of intruding objects.

However, due to the heavy computational burden and difficulty in identifying small targets, the aforementioned algorithms cannot balance the real-time detection performance and accuracy. To achieve good performance, these algorithms have to be run on servers with advanced graphics processing units (GPUs) placed in a data center. This makes these algorithms inappropriate to be applied to practical intrusion detection systems for high-speed railways. Because the sight of the camera mounted on the train can not cover the distance required to stop the high-speed train, in most works, the cameras are installed along the tracks. Therefore, to cover the whole railway lines, a considerable amount of cameras are needed. The cost of the intrusion detection system would not be affordable if the hardware cost for processing the image of each camera was too expensive. In addition, if all the video streams are transmitted to the data center, the network must have a large capacity, which will further increase the cost.

In this study, we proposed an improved deep neural network based on an SSD network for railway intrusion detection, which can achieve good performance on a low-cost embedded GPU. For faster and more powerful intrusion detection, we replaced the VGG-16 in the SSD with a modified MobileNet as the feature extractor. A lightweight and efficient feature fusion model was introduced to fuse the feature maps generated by the deep layers and the shallow layers of the MobileNet. Since the feature maps from deep layers contain more semantic information but have a low resolution to detect small objects, and the feature maps from shallow layers have a higher resolution but less semantic information, the fused features of both deep and shallow feature maps allowed the detection network to improve the performance for small objects. Moreover, depth-wise and point-wise convolution was employed to relieve the computational burden in the feature fusion module. The proposed algorithm was deployed using the TensorRT framework on a low-cost embedded GPU platform, NVIDIA Jetson TX2, to achieve high-performance inference.

## 2. Related Works

Intrusion detection is an important problem for both physical space [20,21,22] and cyberspace [23,24], for safety and security reasons. There are numerous previous studies attempting to address the difficulties of railway clearance intrusion detection. Preexisting methods include the techniques of image processing, machine learning, and CNN-based approaches. Numerous efforts have been made to detect railway intrusion through image processing. Mukojima et al. [25] proposed a background subtraction method for detecting obstacles. First, the method calculated the frame-by-frame correspondence between input and reference train frontal-view camera images. Then, obstacles can be detected by applying image subtraction to the corresponding frame. Tastimur et al. [26] proposed a vision-based method to decrease level crossing accidents. First, YCbCr color transformation, edge extraction, filtering, and Hough transformation were applied to the image to detect horizontal crossing. Then, HSV color transformation, image difference extraction, gradient calculation, filtering detection of connected components, and feature extraction were used for object detection. However, these traditional methods have very poor detection effects in real-time background updates.

Apart from the image-processing method, before deep learning was widely used, many traditional machine learning algorithms were introduced to object detection by researchers. Qi et al. [27] introduced a railway track detection and turnout recognition method to determine the interesting regions for detecting obstacles’ and signals’ histogram of oriented gradients (HOG). Sun and Xie [28] introduced a method for pedestrian intrusion detection of railway perimeter. The method is based on HOG for extracting features and a support vector machine (SVM) for object detection. These methods achieve reasonable detection accuracy in detecting intruding objects and were immune to slight background changes. However, the detecting performance of these methods are substandard when the background changes greatly.

In recent years, object detectors based on deep learning have shown great advantages over traditional methods in difficult object detection situations. The CNN-based detectors can be roughly divided into two categories: one-stage and two-stage. The two-stage approach refers to obtaining candidate boxes by a selective search algorithm first and then predicting the object classification and location. The classical two-stage approaches, such as R-CNN [12], SPP-Net [29], fast R-CNN [13], and faster R-CNN [14], have greatly improved the accuracy of detection upon traditional methods. However, these kinds of methods are difficult to meet real-time requirements due to their computation load and parameters. As a result, one-stage approaches, such as Overfeat [30], YOLO [16], and SSD [17], have gained researchers’ attention. Fu et al. [31] proposed a DSSD model, which replaces the VGG network in the SSD model with the ResNet-101 and applies deconvolution into the feature fusion module to improve the detection performance. However, the computational overhead of said model was also greatly increased. RetinaNet [32] addressed the imbalance of positive and negative anchor boxes during training by proposing a new loss, focal loss, to improve accuracy. RefineDet [33] made full use of the advantages of the one-stage and two-stage methods, thereby reducing the number of negative frames and roughly adjusting the anchor boxes. Moreover, CNN-based object detection algorithms have been widely used in railways. Guo et al. [4] adopted deconvolution to fuse high-level and low-level features for improving the detection performance of small objects. Ye et al. [10] proposed a CNN to detect objects in shunting mode, which consists of three connected modules. Jia et al. [34] studied the advantages and disadvantages of four kinds of object detection algorithms in railway perimeter invasion, which include the unsupervised frame difference method, the background difference method, the supervised class based on deep learning YOLOv3, and faster RCNN. To detect traffic objects on railway tracks, Ye et al. [19] proposed a differential feature fusion neural network, which combines the advantages of the two-stage method and the one-stage method through a prior objection-detection module.

In general, the methods introduced above have their characteristics. Image-processing techniques are sensitive to varying light conditions or complex backgrounds. Machine-learning methods have improved adaptability for the above problems to a certain extent. However, they still cannot cope with any dramatic changes in the background. The CNN-based object detection methods significantly improved the detection accuracy but at a huge computational expense.

The lightweight and efficient model is conducive to the real-time requirement if the machine-learning network is performed on an embedded GPU platform. There are a number of research results about object detection algorithms based on CNN on embedded platforms. Yang et al. [35] proposed an embedded implementation based on SSD for hand detection and orientation estimation, which can detect hands more efficiently on Jetson TK1. Tijtgat et al. [36] proposed a method that applies the YOLOv2 object detection algorithm to an NVIDIA Jetson TX2 to perform real-time object detection onboard an unmanned aerial vehicle (UAV). An implementation of a multiple-faces recognition framework running on the embedded GPU system, NVIDIA Jetson TX2 board, was proposed by [37]. The framework can recognize multiple faces accurately and efficiently. Yudin and Slavioglo [38] proposed a traffic-light detector based on a fully convolutional network and a high-speed clustering method to create the bounding boxes of traffic lights. The test phase of the network was implemented on an embedded system based on NVIDIA Jetson TX2.

## 3. The Proposed Network Structure

**Figure 1:**
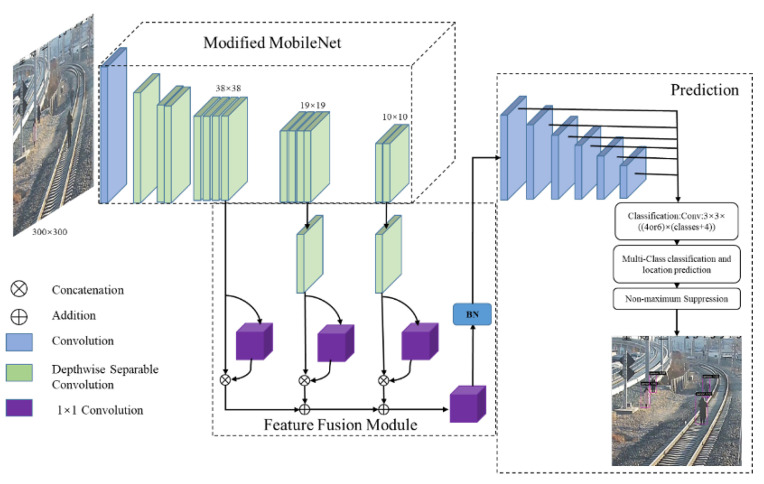
The structure of the proposed network, which mainly consists of a modified MobileNet to extract three feature maps of different scales, and a feature fusion module to merge the context information and the detailed features to detect the small objects.

Our proposed method aims to improve the detection performance of small intruding objects and meet the real-time detection requirements on low-cost embedded GPUs. The architecture of the proposed network is shown in Figure 1. The network mainly consists of two parts: the feature extraction module and the feature fusion module. The feature extraction network was constructed with MobileNet, which was pre-trained on ImageNet. The feature extraction module is responsible for extracting features of the object in the input image, which generates three feature maps of different scales as the input to the feature fusion module. The main purpose of the feature fusion module is to capture both the local detailed features and global semantic features from multiple feature maps and merge the context information and the detailed features to detect the small objects. Upon the end of said procedures, same as with the conventional SSD, the pyramid feature map composed of several simple blocks is adapted to generate the bounding boxes for different scales, with their locations and categories. Finally, the location of the bounding box is refined by a non-maximum suppression algorithm to obtain the final detection results.

### 3.1. Feature Extraction Module

**Figure 2:**
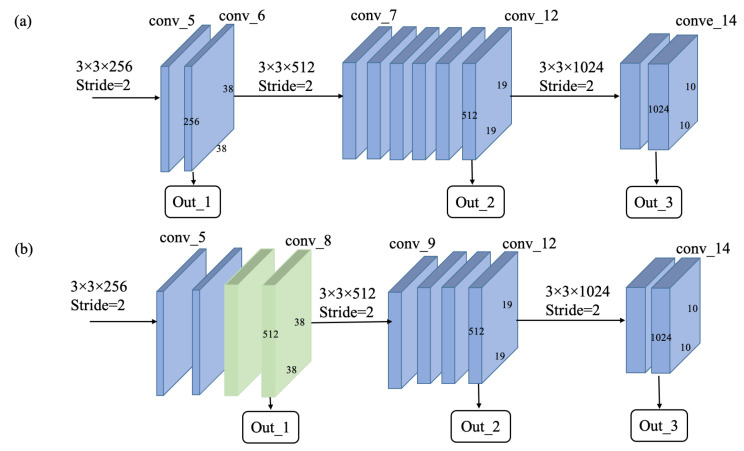
The architecture of the improved MobileNet: (**a**) the original MobileNet and (**b**) the improved MobileNet, which doubles the resolutions of lower layers to improve the detection performance of small objects.

With the trade-off of the accuracy and the real-time performance on an embedded device in mind, we replaced the VGG-16 with the MobileNet to reduce the computational complexity of the CNN-based network. For object detectors based on ConvNets, the low-level feature map is responsible for detecting small-scale objects, whereas the high-level feature map corresponds to large-scale object detection. As shown in Figure 2a, the output layers of the original MobileNet as a feature extractor of the SSD framework are conv_6, conv_12, and conv_14. These layers are more suited for locating and recognizing large objects instead of smaller objects due to the loss of high-resolution information. Therefore, some modifications, as shown in Figure 2b, were made to improve the detection performance of small objects. Based on the original MobileNet, the stride of the depth-wise convolution of conv_7 was changed from 2 to 1. Then, the output of each subsequent layer will maintain the same resolution of 38 × 38 before conv_9, in which it was down-sampled to 19 × 19, and the stride of conv_9 was changed to 2 instead of 1 in previous layers. For the improved MobileNet, the feature layer of conv_8 (size of 38 × 38) was used to detect small objects, and conv_14 (size of 10 × 10) corresponds to detection for large objects. The doubled resolutions of conv_7 and conv_8 do not increase too much the computational burden while largely improving the accuracy of object detection compared to the original MobileNet. In this study, feature layers of conv_8 (size of 38 × 38 with 512 channels), conv_12 (size of 19 × 19 with 512 channels), and conv_14 (size of 10 × 10 with 1024 channels) were considered to be the outputs of the feature extraction network. Such a set of feature maps with various resolutions were provided as input to the feature fusion module.

### 3.2. Adaptive Feature Fusion Module

**Figure 3:**
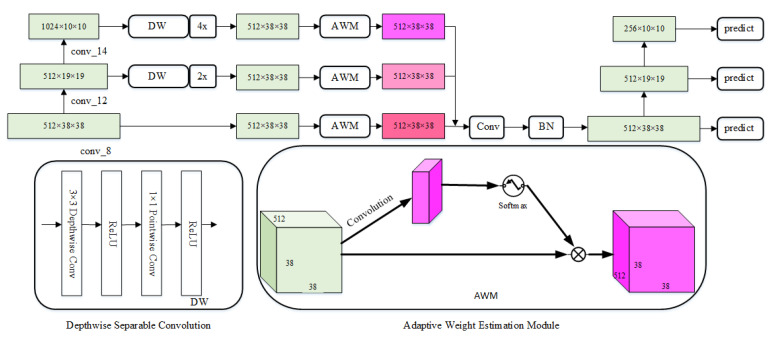
The adaptive feature fusion (AFF) module, which integrates context information from three different levels of feature maps adaptively.

The feature fusion method is widely used in object detection algorithms to improve the performance of the conventional detectors. In our study, we introduced an efficient adaptive feature fusion (AFF) module (shown in Figure 3) to integrate context information from three different levels of feature maps (conv_8, conv_12, conv_14) adaptively. Since the dimensions of the feature maps are different, the AFF module first transforms the dimensions of conv_12 and conv_14 to the same dimension of conv_8. To do this, the depth-wise and point-wise convolution is applied to transform the numbers of feature map channels of conv_12 and conv_14 to 512, and their spatial dimensions are up-sampled by the bilinear interpolation to 38×38. As a result, all the feature maps have the same size on all dimensions.

Since different feature maps have different contributions to the final accuracy of object detection, an adaptive weight estimation module was introduced to learn the spatial weight of feature maps at each scale. The feature vectors at level *l* (l∈1,2,3 corresponding to conv_8, con_12, and con_14 respectively) are referred to as $x^{l}$. The spatial importance weights of the three different levels of feature maps are represented by α, β, and γ, respectively. Specifically, we forced α+β+γ=1 and α,β,γ∈[0,1], which can be calculated by:

(1) $$\begin{array}{c} \alpha =\frac{e^{\lambda \alpha }}{e^{\lambda \alpha }+e^{\lambda \beta }+e^{\lambda \gamma }},\\ \beta =\frac{e^{\lambda \beta }}{e^{\lambda \alpha }+e^{\lambda \beta }+e^{\lambda \gamma }},\\ \gamma =\frac{e^{\lambda \beta }}{e^{\lambda \alpha }+e^{\lambda \beta }+e^{\lambda \gamma }}, \end{array}$$

where α, β, and γ are defined by using the softmax function with $\lambda_{\alpha }$, $\lambda_{\beta }$, and $\lambda_{\gamma }$ as control parameters, respectively. The weight adaption layer was used to compute the weighted scalar graphs $\lambda_{\alpha }$, $\lambda_{\beta }$, and $\lambda_{\gamma }$ from $x^{1}$, $x^{2}$, and $x^{3}$, respectively. Then, the sum of $\alpha x^{1}$, $\beta x^{2}$ and $\gamma x^{3}$ were taken as the input to a 1×1 convolution to expand the number of channels to 1024, and a batch normalization (BN) layer was accepted to normalize the feature values. Finally, some down-sampling blocks were appended to generate a new feature pyramid to produce object-detection results.

### 3.3. Network Training

Using the railway data as the experimental object, the network was trained for 300 epochs, with a starting learning rate of 0.001, 0.9 momentum, 0.0005 weight decay, and batch size 32. After 100 epochs, an exponential learning rate decay policy was applied. For a fair comparison, the same matching strategy, hard negative mining strategy, and data augmentation as the SSD were adopted. Additionally, the joint localization loss and confidence loss were minimized by the smooth L1 and the cross-entropy function, respectively.

### 3.4. Implementation and Optimization on Embedded GPUs

We deployed the proposed network on the NVIDIA Jetson TX2 module, which has an NVIDIA Pascal-family GPU with 256 NVIDIA CUDA cores. It also has a very low power consumption (about 10 W), which is important for embedded solutions. The Jetson TX2 runs under the operating system Ubuntu 16.04.

In order to reduce computation and model size, we applied the depth-wise separable convolution, which is a form of factorized convolutions, which factorize a standard convolution into a depth-wise convolution and a point-wise convolution. The depth-wise convolution applies a single filter to each input channel, and then the point-wise convolution applies a 1×1 convolution to create a linear combination of the output of the depth-wise layer. By using 3×3 depth-wise separable convolutions, we can gain eight to nine times less computation than standard convolution under only a small reduction in accuracy.

To accelerate the inference, the TensorRT framework was used to deploy the network. TensorRT is a neural network inference framework, which is optimized to accelerate the inference speed and to reduce the model size, and it can achieve significant computing efficiency for deploying neural networks on marginal computing devices. We first created and trained our network with the Pytorch framework on a desktop with an NVIDIA GTX 1060 GPU. Next, the network model created by the Pytorch framework was converted to the open neural network exchange (ONNX) format and then transformed into the TensorRT format using the ONNX parser, which is an efficient and popular method to obtain a deployment-ready run-time inference engine. Finally, the converted TensorRT format can be deployed to the Jetson TX2.

## 4. Experimental Results

The proposed method was comprehensively evaluated on a railway-intrusion detection dataset and the public PASCAL VOC datasets to verify the effectiveness. To study the performance of the proposed method, the criterion of average precision (AP) was introduced, which depends on precision and recall. In addition, several experiments were performed on the classic one-stage approach SSD, YOLOv2, and the two-stage Faster R-CNN to compare the efficiency of our approach. All experiments were conducted on the deep learning framework Pytorch, and the effects of different structural designs were examined in an ablation study.

### 4.1. Datasets

To evaluate the performance for railway intrusion detection of the proposed methods, we created a railway intrusion detection dataset. To ensure the diversity in our railway data, a series of railway traffic videos were collected from the several actual high-speed railway lines under a number of distinct conditions of weather and lighting. From the videos, 17,078 image frames were sampled, containing all frames with intruding objects and background frames sampled every second in the video. Intruding objects in the frames were labeled with two classes: pedestrian and train. (Trains were regarded as normal at the post-processing stage.) We shuffled 70% of the dataset for training and validation, and the remaining data were used for testing.

PASCAL VOC datasets were also used to compare the performance with other methods. VOC datasets provide standardized-image datasets for object category recognition, which contain 20 categories. The datasets of VOC 2007 trainval and 2012 trainval were treated as train datasets, and the VOC 2007 test dataset was adopted to evaluate the detection performance.

### 4.2. Experiment on NVIDIA Jetson TX2

We first evaluated the intrusion detection accuracy and processing time of the proposed method. To compare the performance, we implemented the network on both a desktop GPU (GTX 1060) and an embedded GPU (Jetson TX2), which were evaluated on the railway intrusion dataset. The results are listed in Table 1. The mAP was used to indicate the accuracy, and the average time for processing a frame was also given to evaluate the processing speed.

In the table, FP32 and FP16 denote that TensorRT store weights and execution layers in 32-bit and 16-bit floating-point numbers, respectively. By comparing the performance for Pytorch and TensorRT on the GTX 1060 platform, we can see that the TensorRT framework was faster with a slight decrease in accuracy. When using the embedded platform (Jetson TX2), the model achieved similar accuracy with the model deployed on the desktop platform, although the processing time increased significantly due to the limited computation resources of the TX2. However, we found that if the computation precision was set to be FP16, the processing speed increased dramatically, while the mAP remained the same with FP32 precision.

**Figure 4:**
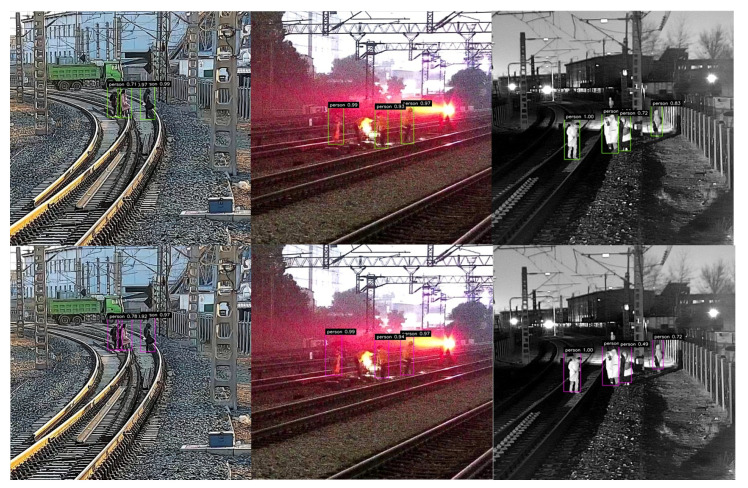
The visual results of the proposed network under different devices and deep learning frameworks. Top row: results of the network implemented on the NVIDIA GTX 1060; bottom row: results of the network implemented on the Jetson TX2.

Figure 4 shows the detection performance under different devices and deep learning frameworks on the railway dataset. The top row represents the performance of our method trained on the deep learning framework Pytorch with the NVIDIA ® GTX® 1060. The bottom row is the detection results performed on the Jetson TX2 with the TensorRT framework using the precision of FP16. From the comparison results, it can be seen that the confidence predicted by the TensorRT framework was slightly reduced, but the detection performance was not affected, which demonstrates the potential for the proposed method to be well-applied to the detection of railway intrusion objects under the embedded platform.

### 4.3. Ablation Study

To further understand the effects of the feature extraction module and the feature fusion module, the experiment results shown in Table 2 of our network under different designs were analyzed in the PASCAL VOC datasets. Taking the improved MobileNet as the feature extractor, a 2.45 percent AP improvement was achieved based on a fundamental architecture combing the SSD and the original MobileNet, and, by introducing the feature fusion module, the precision was further increased by 2.62 percent. The experimental results in row 2 and row 4 illustrate the efficiency of the feature fusion module, especially when the feature extraction network was more powerful. Therefore, these techniques are essential for improving the detection performance of intruding objects.

### 4.4. Performance of Small-Object Detection

**Figure 5:**
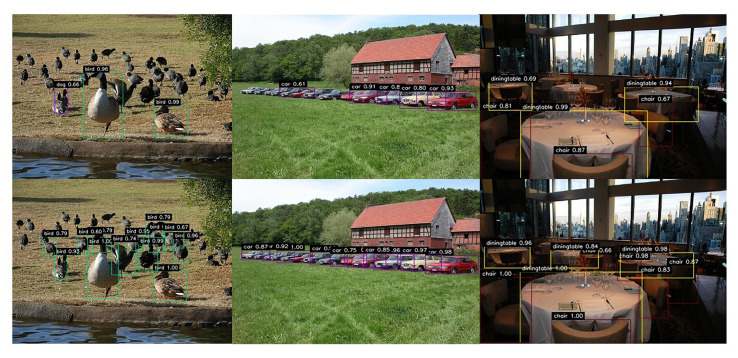
Comparison of detection results of small objects between the original SSD (**top row**) and the proposed method (**bottom row**).

In this section, we demonstrate the improvements of our proposed method on small-object detection in the PASCAL VOC datasets. The comparison results between the original SSD and the proposed network in detecting small objects are shown in Figure 5. The following assumptions were made to evaluate the performance of the proposed network for small-object detection on PASCAL VOC datasets. First, regarding the size of the network input, we defined objects that had an area less than 1024 as small objects. Next, if the IOU between the bounding box and the ground truth box was greater than 0.4 s, it was treated as a positive sample. Based on the above assumptions, 2933 small samples were selected from the VOC 2007 test dataset. The experimental results show that the proposed method can detect 517 samples from the small-object dataset, which was 238 more than the SSD. This difference demonstrates that our method improves upon the original SSD in detecting small objects.

Moreover, the visual detection results on the PASCAL VOC datasets are shown in Figure 5. The top row represents the performance of the original SSD. The bottom row is the detection results of the proposed method. In the first column, our method detected two more birds compared to the original SSD. In the second column, four more cars were detected by our method. In the third column, our method detected three more chairs and two more dining tables. Meanwhile, the confidence of almost all categories was improved notably in the proposed method.

### 4.5. Comparison with State-of-the-Art Methods

The experimental results compared with the other three state-of-art networks are demonstrated in Table 3. The detection performance of the proposed network was better than faster R-CNN, YOLOv2, and SSD by 2.9%, 2.4%, and 1.8%, respectively. In addition, the FLOPs inference time of each network was investigated. For fair comparisons, the inference time was evaluated 100 times with a batch size of 1 on a machine equipped with an NVIDIA GTX® 1060, CUDA 9.0, and cuDNN v7. As shown in Table 3, the processing speed of each image was higher than the speed of the other three networks. Compared with faster R-CNN, YOLOv2, and SSD, the computational burden of the proposed method was significantly reduced.

The comparison results in the railway dataset are shown in Table 4. With an approximate input size of 300×300, the proposed method can achieve an mAP of 89.66%, which is much better than the other three state-of-art networks. The experimental results of the faster R-CNN were relatively good due to its ability to fully extract features from large images and because the image size in the railway dataset was close to the input size of the faster R-CNN.

**Figure 6:**
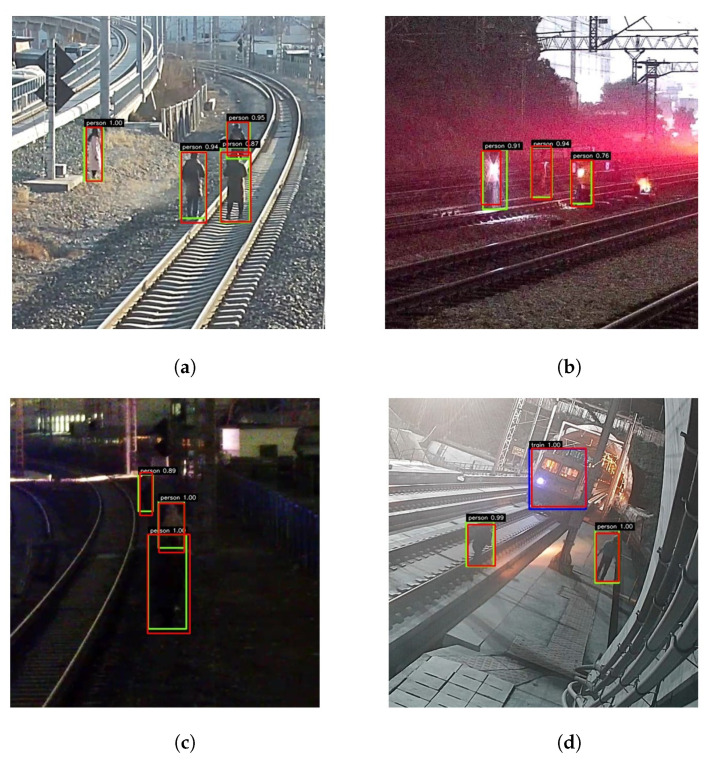
The performance of our method in different environmental conditions. (**a**) Pedestrian on railway track. (**b**) Pedestrian in the light. (**c**) Pedestrian at night. (**d**) Pedestrian and train.

Figure 6 shows the robustness of the proposed method under different environmental conditions. The red box represents the ground-truth label for each object. The detection performance of pedestrians and trains is indicated by yellow and blue boxes. Figure 6a shows that the proposed method can detect pedestrians crossing the railway with high classification scores and good location regression on sunny days. As shown in Figure 6b,c, the proposed method can detect obstacles well in both night and light environments to ensure the operational safety of the train. Figure 6d illustrates our attempt to determine the existence of intruding objects on a specific railway where a train is passing by. The detection performance under different environments, especially with low-quality images caused by bad weather, indicates that the proposed method can meet the requirements of practical applications.

## 5. Conclusions

In this study, a lightweight CNN network based on SSD was proposed to meet the requirements of an embedded GPU platform in railway applications. The improved MobileNet was introduced to replace VGG-16 as a feature extractor to relieve computational complexity. A novel feature fusion module was presented to further introduce context information that benefits small-object detection. The experimental results on the railway dataset have demonstrated that the proposed method achieved better performance for railway-intrusion detection compared with three other state-of-the-art object-detection methods. The detection performance of small objects on PASCAL VOC datasets indicated that our method is superior to the conventional SSD. In addition, the proposed method achieved good performance in different environmental conditions of the railway. Finally, the TensorRT framework was adopted to deploy our method into the embedded GPU system, NVIDIA Jetson TX2 board, which significantly decreased the inference time. The experiment on the NVIDIA Jetson TX2 module showed that the proposed methods can be deployed on a low-cost embedded GPU platform to achieve good performance and a decent processing speed. We demonstrated a low-cost solution to intrusion detection for high-speed railways, which makes the long-range intrusion detection system for high-speed railways affordable. As a low-cost embedded solution, it may also be used in on-board obstacle detection of subway and urban rails.

## Figures and Tables

**Table 1:** Acceleration results of the proposed method.

Method	Platform	Framework	Precision	mAP (%)	Time (ms)
Our Method	GTX® 1060	Pytorch	FP32	89.66	14.30
	GTX® 1060	TensorRT	FP32	89.53	9.20
	Jetson TX2	TensorRT	FP32	89.60	53.95
	Jetson TX2	TensorRT	FP16	89.60	35.63

**Table 2:** The test detection performance of networks under different design choices.

MobileNet	Improved MobileNet	Feature Fusion	mAP (%)
✓			71.06
✓		✓	73.62
	✓		73.51
	✓	✓	76.13

**Table 3:** PASCAL VOC 2007 test detection results.

Method	Backbone	Input Size	FLOPs (M)	mAP (%)	Time (ms)
Faster R-CNN	VGG-16	1000×600	149,200	73.2	114.4
YOLOv2	DarkNet-19	352×352	10,551	73.7	15.2
SSD300	VGG-16	300×300	31,339	74.3	21.0
Our method	MobileNet	300×300	11,850	76.1	14.5

**Table 4:** Comparison results of different algorithms on railway datasets.

Method	mAP (%)	Pedestrian	Train	FLOPs (M)	Time (ms)
Faster R-CNN	89.55	89.61	89.49	149,090	104.7
YOLOv2	82.86	78.52	87.20	10,544	15.0
SSD300	88.18	88.80	87.55	30,503	20.9
Our method	89.66	89.75	89.58	10,919	14.3

## Data Availability

Publicly available datasets were analyzed in this study. This data can be found here: http://host.robots.ox.ac.uk/pascal/VOC/ (accessed on 1 November 2021).
